# Exploring weight data on over 100,000 Swedish dogs of various breeds

**DOI:** 10.1186/1751-0147-57-S1-O8

**Published:** 2015-09-25

**Authors:** Linda Andersson, Marcin Kierczak, Veronika Scholz, Janet Johansson, Åke Hedhammar

**Affiliations:** 1 The Swedish Kennel Club, Stockholm, Sweden; 2 Science for Life Laboratory, Department of Medical Biochemistry and Microbiology, Uppsala University, Uppsala, Sweden; 3 Department of Clinical Sciences, Swedish University of Agricultural Sciences, Uppsala, Sweden

## Objectives

This study aims to explore a unique resource of weight and health data for further studies on effects of body weight and body condition score on developmental degenerative and metabolic diseases in dogs.

## Methods

Our study-data on weight at 12 months of age for more than 100,000 dogs of various breeds from the dog database at the Swedish Kennel Club (SKK) have enabled us to:

– provide a reference for further studies by characterizing distribution of adult body weights in various breeds, also stratified by other factors, e.g. sex.

– explore how weight influences hip and elbow status recorded in the same database.

– investigate how to combine these data with recording of body condition score and to merge them with diagnostic information as insurance claim data.

## Results

There is a wide range of body weights within all analyzed breeds as well as between the sexes within breeds. There is a pronounced correspondence between body weight at 12 months of age and radiological status of hips and elbows. It is also possible to include body condition score in further analyses and also to merge the data with anonymized information from an insurance claim database.

## Conclusion

We showed that the available body weight data are well-suited for further studies on the role and effects of both weight and body condition score in various diseases in dogs.

